# Supplementary material for: High-Pressure Depolymerization of Poly(lactic acid) (PLA) and Poly(3-hydroxybutyrate) (PHB) Using Bio-Based Solvents: A Way to Produce Alkyl Esters Which Can Be Modified to Polymerizable Monomers
Source: Polymers (Basel). 2022 Dec 1;14(23):5236. doi: 10.3390/polym14235236 (PMC9739185; doi:10.3390/polym14235236)
Supplement: Supplementary file 1 [file polymers-14-05236-s001.zip › polymers-2052066-supplementary.pdf]

Supplementary material for article: polymers–2052066

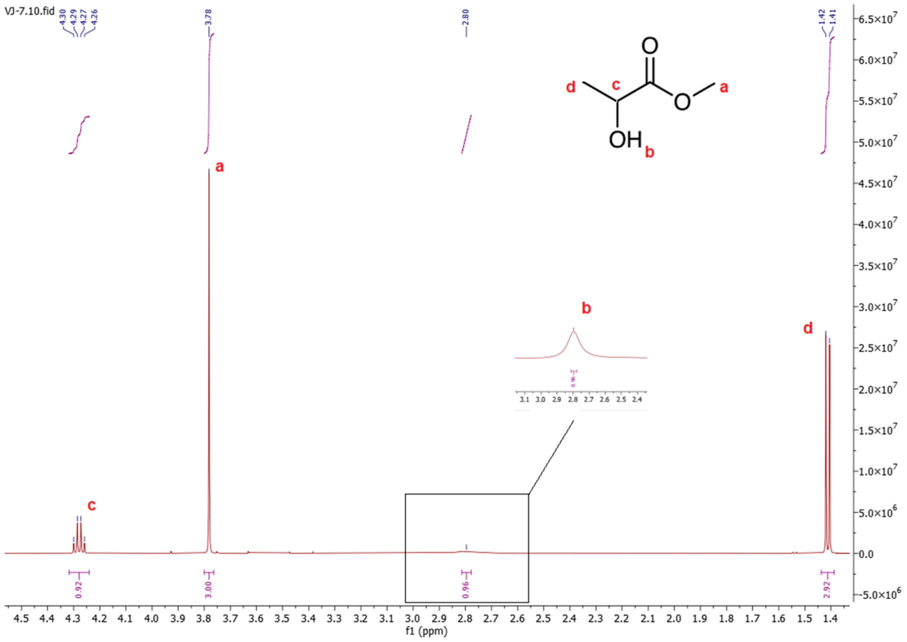

Figure S1. ^{1}H NMR spectrum of methyl lactate (MeLa). ^{1}H NMR ($CDCl_3$, 500 MHz): δ (ppm) 4.30–4.26 (q; *J* = 6.9 Hz; 1H), 3.78 (s; 3H), 2.80 (s; 1H), 1.42–1.41 (d; *J* = 6.9 Hz; 3H).

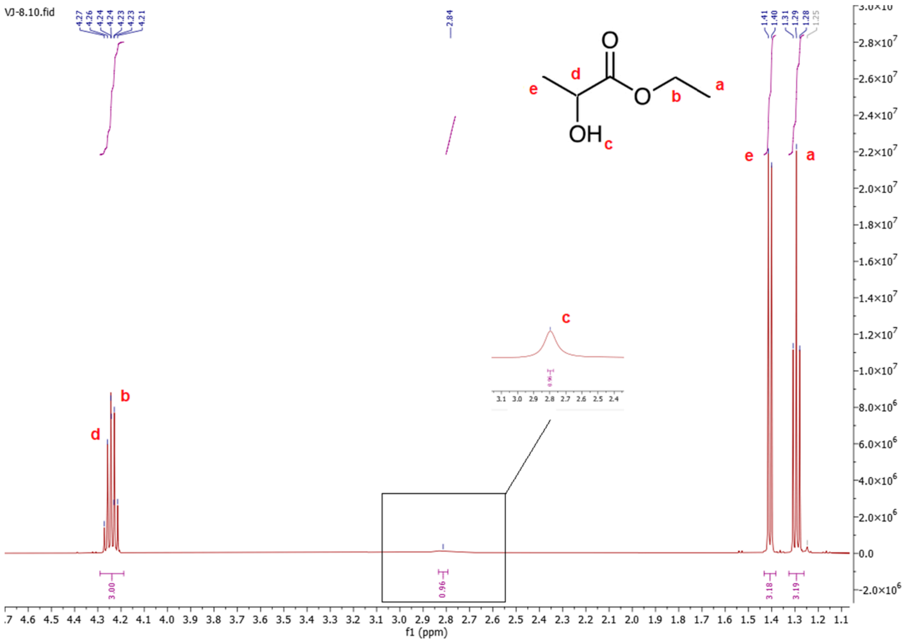

Figure S2. ^{1}H NMR spectrum of ethyl lactate (EtLa). ^{1}H NMR ($CDCl_3$, 500 MHz): δ (ppm) 4.27–4.21 (m; 3H), 2.84 (s; 1H), 1.41–1.40 (d; *J* = 6.9 Hz; 3H), 1.31–1.28 (t; *J* = 7.16; 7.16 Hz; 3H).

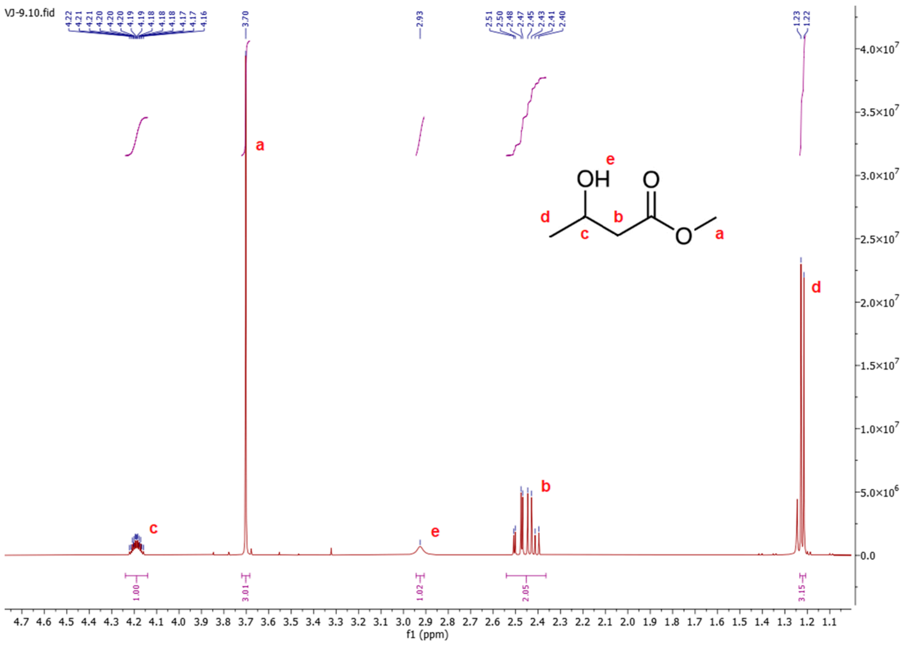

Figure S3. ^{1}H NMR spectrum of methyl 3–hydroxybutyrate (M3HB). ^{1}H NMR ($CDCl_3$, 500 MHz): δ (ppm) 4.22–4.16 (qd; *J* = 8.54; 6.30; 6.27; 6.27 Hz; 1H), 3.70 (s; 3H), 2.93 (s; 1H), 2.51–2.40 (m; 2H), 1.23–1.22 (d; *J* = 6.30 Hz 3H).

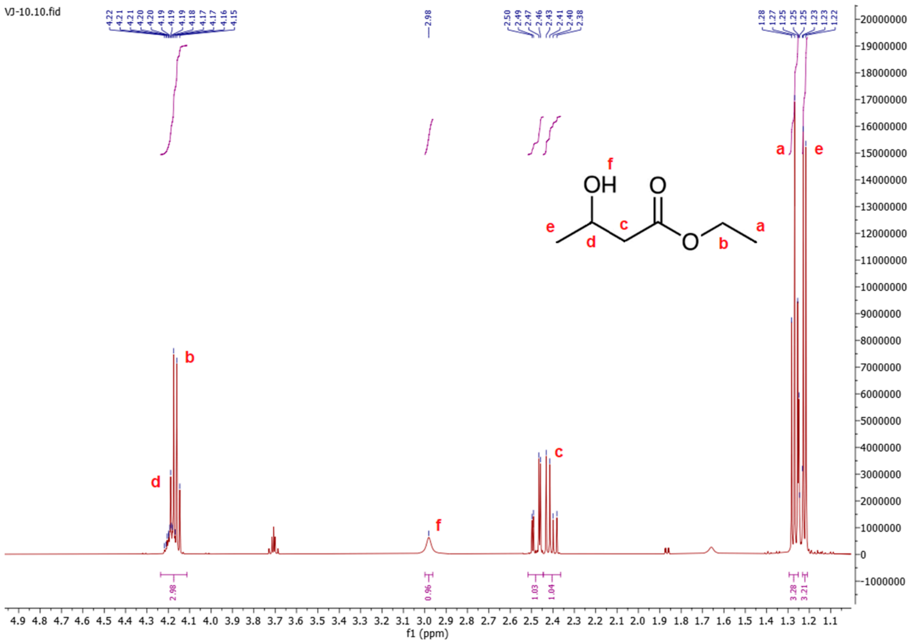

Figure S4. ^{1}H NMR spectrum of ethyl 3–hydroxybutyrate (E3HB). ^{1}H NMR ($CDCl_3$, 500 MHz): δ 4.22–4.15 (q; *J* = 7.16; 7.12; 7.12 Hz; 3H), 2.98 (s; 1H), 2.50–2.46 (dd; *J* = 16.38; 3.48 Hz; 1H), 2.43–2.38 (dd; *J* = 16.40; 8.68 Hz; 1H), 1.28–1.25 (t; *J* = 6.52; 6.52 Hz; 3H), 1.23–1.22 (d; *J* = 6.93 Hz; 3H).

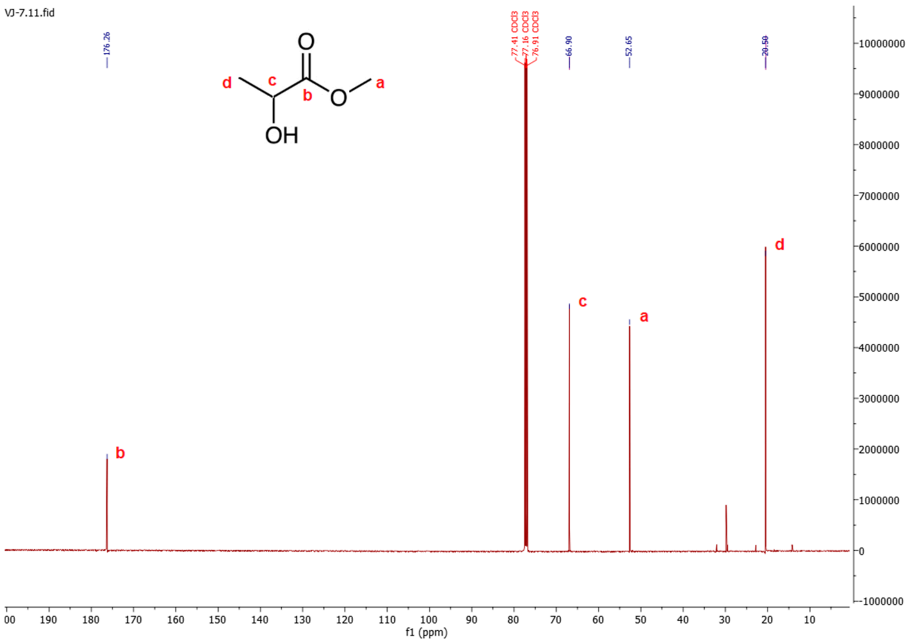

Figure S5. ^{13}C NMR spectrum of methyl lactate (MeLa). ^{13}C NMR ($CDCl_3$, 126 MHz): δ (ppm) 176.26; 66.90; 52.65; 20.50.

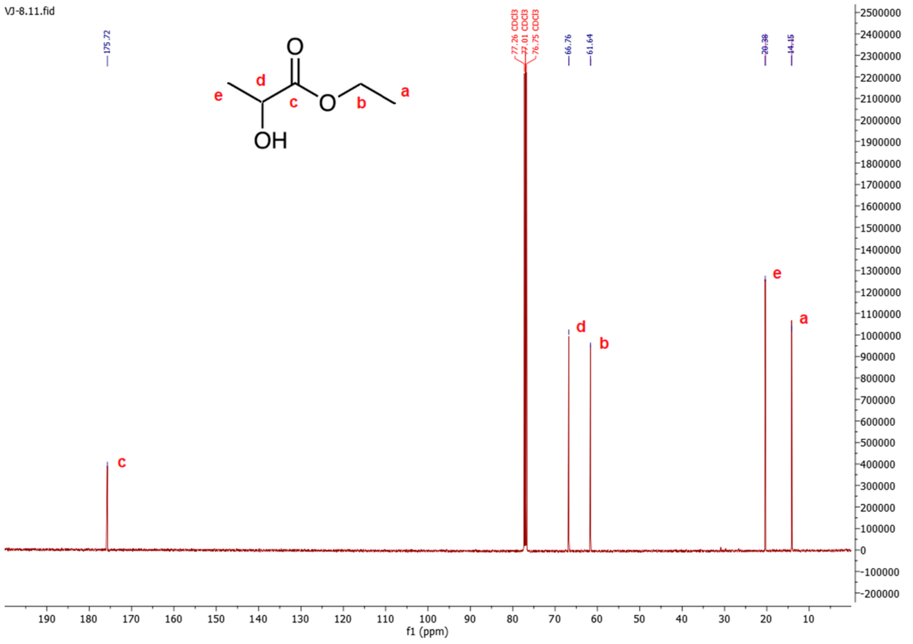

Figure S6. ^{13}C NMR spectrum of ethyl lactate (EtLa). ^{13}C NMR ($CDCl_3$, 126 MHz): δ (ppm) 175.72; 66.76; 61.64; 20.38; 14.15.

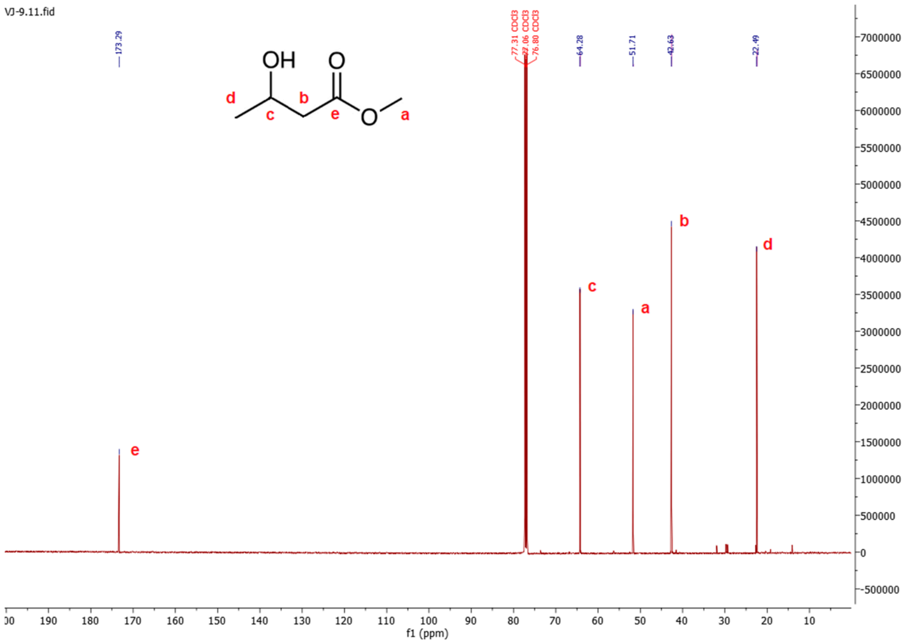

Figure S7. ^{13}C NMR spectrum of methyl 3–hydroxybutyrate (M3HB). ^{13}C NMR ($CDCl_3$, 126 MHz): δ (ppm) 173.29; 64.28; 51.71; 42.63; 22.49.

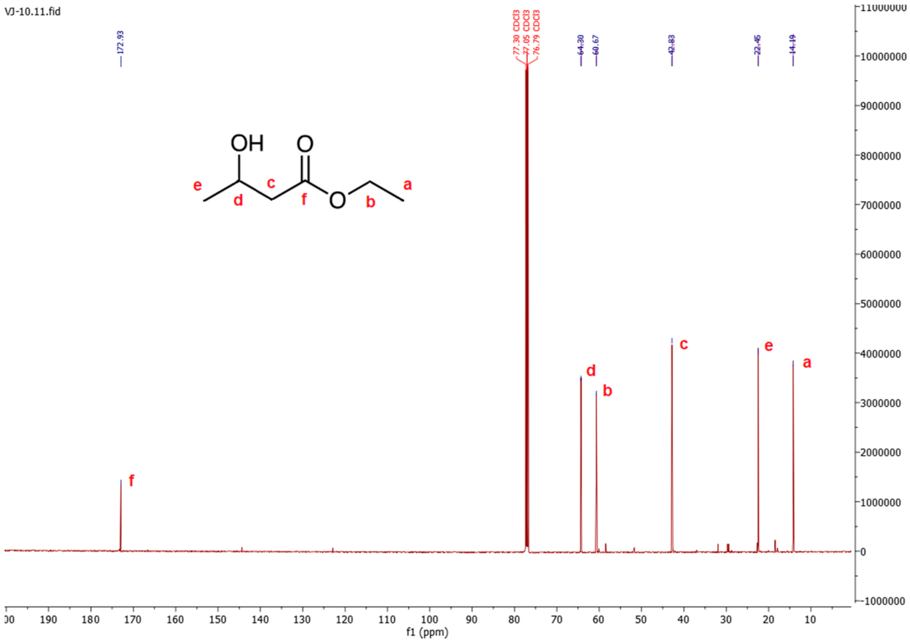

Figure S8. ^{13}C NMR spectrum of ethyl 3–hydroxybutyrate (E3HB). ^{13}C NMR ($CDCl_3$, 126 MHz): δ (ppm) 172.93; 64.30; 60.67; 42.83; 22.45, 14.19.

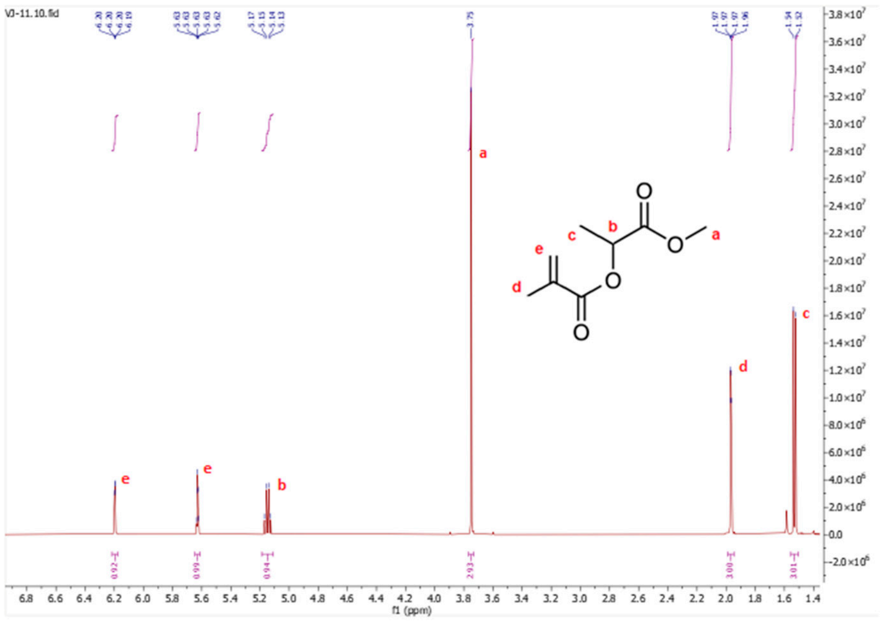

Figure S9. ^{1}H NMR spectrum of methacrylated methyl lactate (MeLaMMA). ^{1}H NMR ($CDCl_3$, 500 MHz): δ (ppm) 6.20–6.19 (dd; *J* = 1.54; 0.94 Hz; 1H), 5.63–5.62 (p; *J* = 1.53; 1.53; 1.52; 1.52 Hz; 1H), 5.17–5.13 (q; *J* = 7.08; 7.07; 7.07 Hz; 1H), 3.75 (s; 3H), 1.97–1.96 (dd; *J* = 1.6; 1.00 Hz; 3H), 1.53 (d; *J* = 7.00 Hz; 3H).

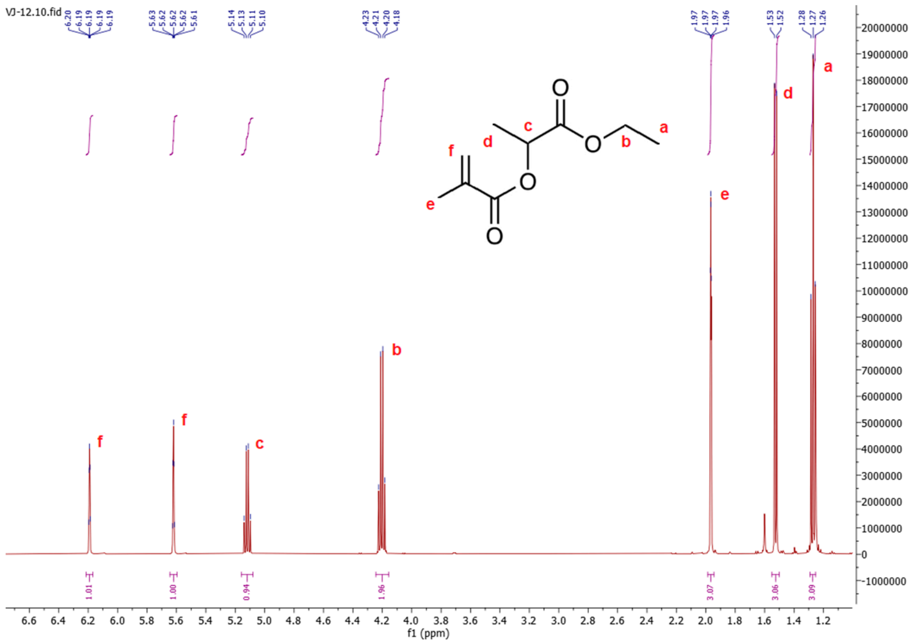

Figure S10. ^{1}H NMR spectrum of methacrylated ethyl lactate (EtLaMMA). ^{1}H NMR ($CDCl_3$, 500 MHz): δ (ppm) 6.20–6.19 (p; *J* = 1.07; 1.07; 1.07; 1.07 Hz; 1H), 5.63–5.61 (p; *J* = 1.57; 1.57; 1.57; 1.57 Hz; 1H), 5.14–5.10 (q; *J* = 7.07; 7.07; 7.03 Hz; 1H), 4.23–4.18 (q; *J* = 7.16; 7.16; 7.15 Hz; 2H), 1.97–1.96 (dd; *J* = 1.58; 1.01 Hz; 3H), 1.53–1.52 (d; *J* = 7.05 Hz; 3H), 1.28–1.26 (t; *J* = 7.15; 7.15 Hz; 3H).

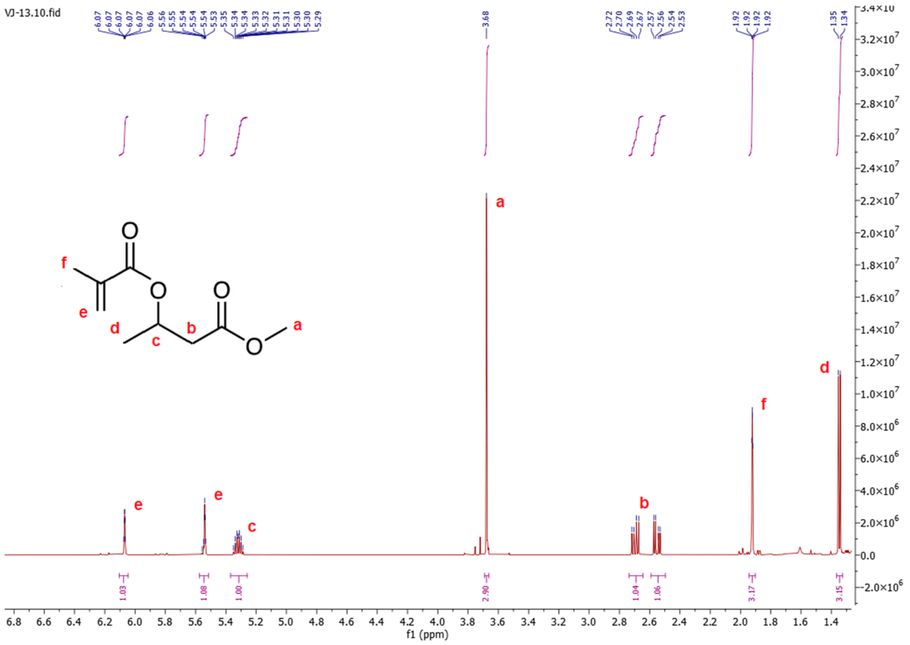

Figure S11. ^{1}H NMR spectrum of methacrylated methyl 3–hydroxybutyrate (M3HBMMA). ^{1}H NMR ($CDCl_3$, 500 MHz): δ (ppm) 6.07–6.06 (dq; J = 1.96; 1.02; 0.98; 0.98 Hz; 1H), 5.56–5.53 (p; J = 1.60; 1.60; 1.58; 1.58 Hz; 1H), 5.35–5.29 (dp; J = 7.32; 6.26; 6.26; 6.25; 6.25 Hz; 1H), 3.68 (s; 3H), 2.72–2.67 (dd; J = 15.34; 7.29 Hz; 1H), 2.57–2.53 (dd; J = 15.35; 5.79 Hz; 1H), 1.92 (dd; J = 1.63; 1.01 Hz; 3H), 1.35–1.34 (d; J = 6.36 Hz; 3H).

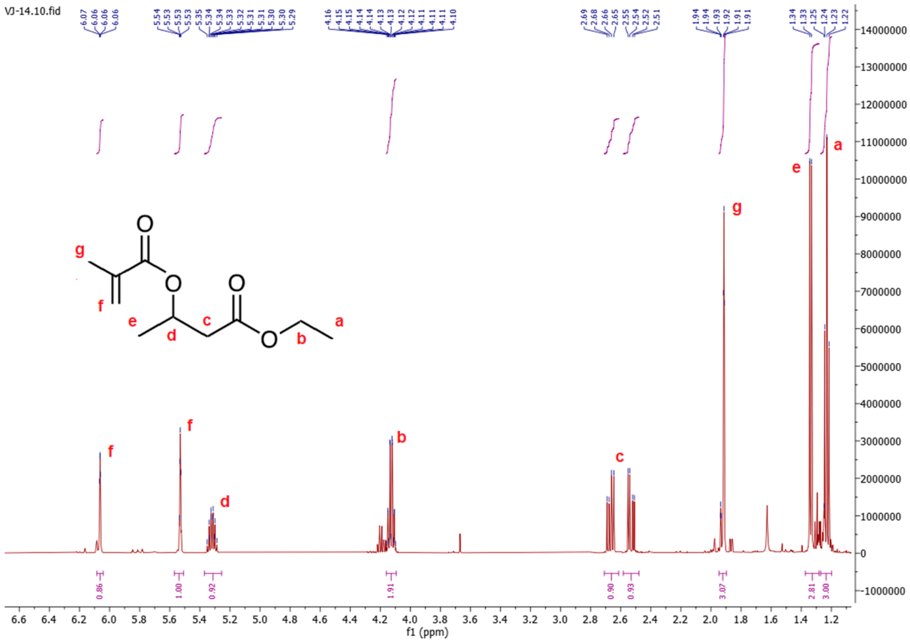

Figure S12. ^{1}H NMR spectrum of methacrylated ethyl 3–hydroxybutyrate (E3HBMMA). ^{1}H NMR ($CDCl_3$, 500 MHz): δ (ppm) 6.07–6.06 (dd; J = 1.75; 0.97 Hz; 1H), 5.54–5.53 (q; J = 1.63; 1.63; 1.63 Hz; 1H), 5.35–5.29 (dp; J = 7.50; 6.24; 6.24; 6.24; 6.24 Hz; 1H), 4.16–4.10 (qd; J = 7.11; 7.06; 7.06; 0.96 Hz; 2H), 2.69–2.65 (dd; J = 15.28; 7.42 Hz; 1H), 2.55–2.51 (dd; J = 15.29; 5.75 Hz; 1H), 1.94–1.91 (m; 3H), 1.34–1.33 (d; J = 6.28 Hz; 3H), 1.25–1.22 (t; J = 7.13; 7.13 Hz; 3H).

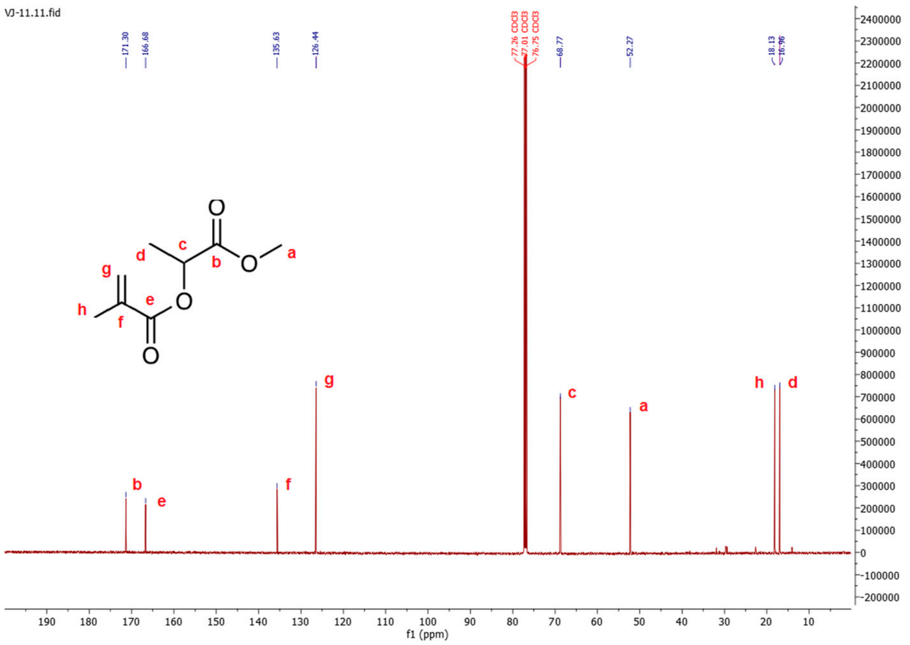

Figure S13. ^{13}C NMR spectrum of methacrylated methyl lactate (MeLaMMA). ^{13}C NMR ($CDCl_3$, 126 MHz): δ (ppm) 171.30; 166.68; 135.63; 126.44; 68.77; 52.27; 18.13; 16.96.

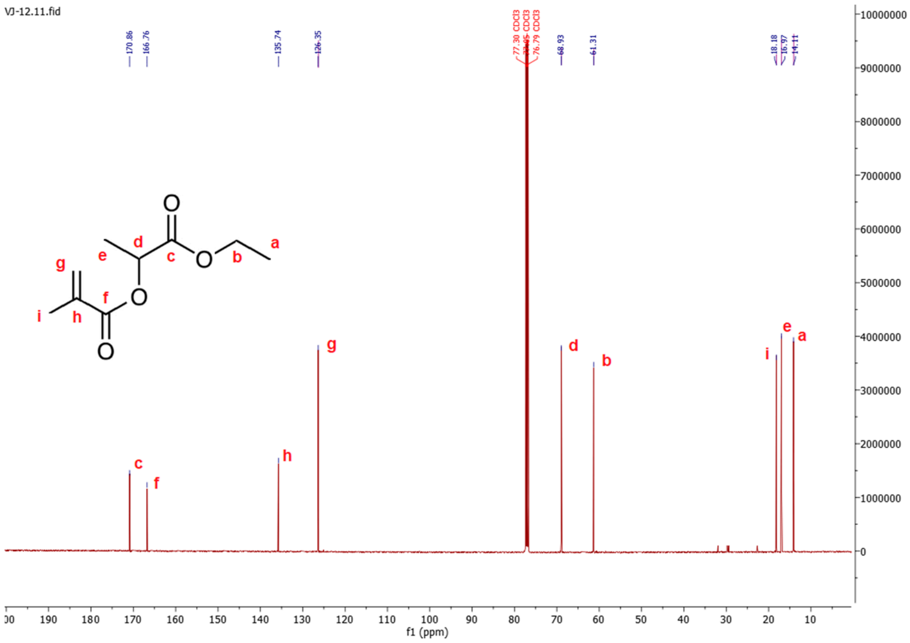

Figure S14. ^{13}C NMR spectrum of methacrylated ethyl lactate (EtLaMMA). ^{13}C NMR ($CDCl_3$, 126 MHz): δ (ppm) 170.86; 166.76; 135.74; 126.35; 68.93; 61.31; 18.18; 16.97; 14.11.

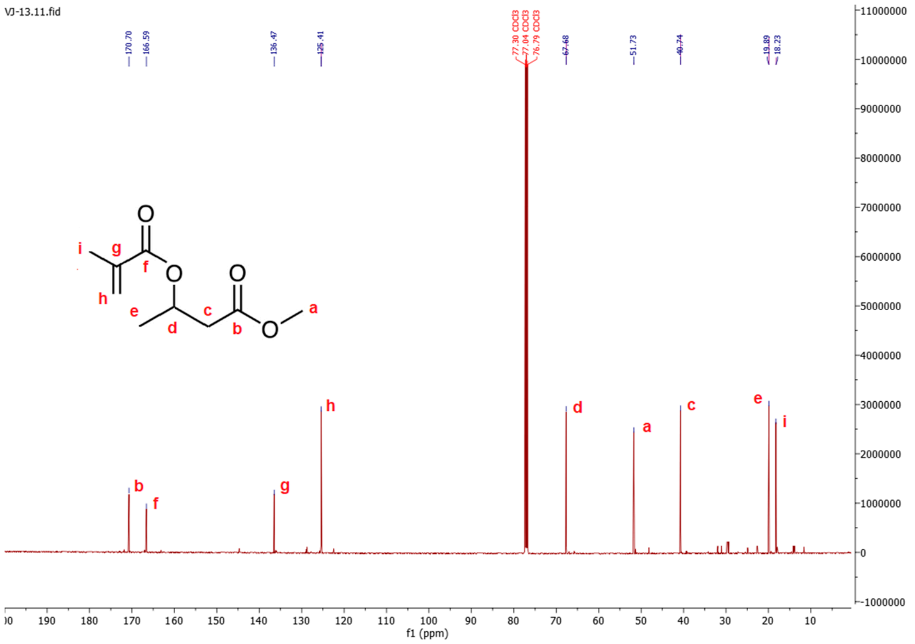

Figure S15. ^{13}C NMR spectrum of methacrylated methyl 3–hydroxybutyrate (M3HBMMA). ^{13}C NMR ($CDCl_3$, 126 MHz): δ (ppm) 170.70; 166.59; 136.47; 125.41; 67.68; 51.73; 40.74; 19.89; 18.23.

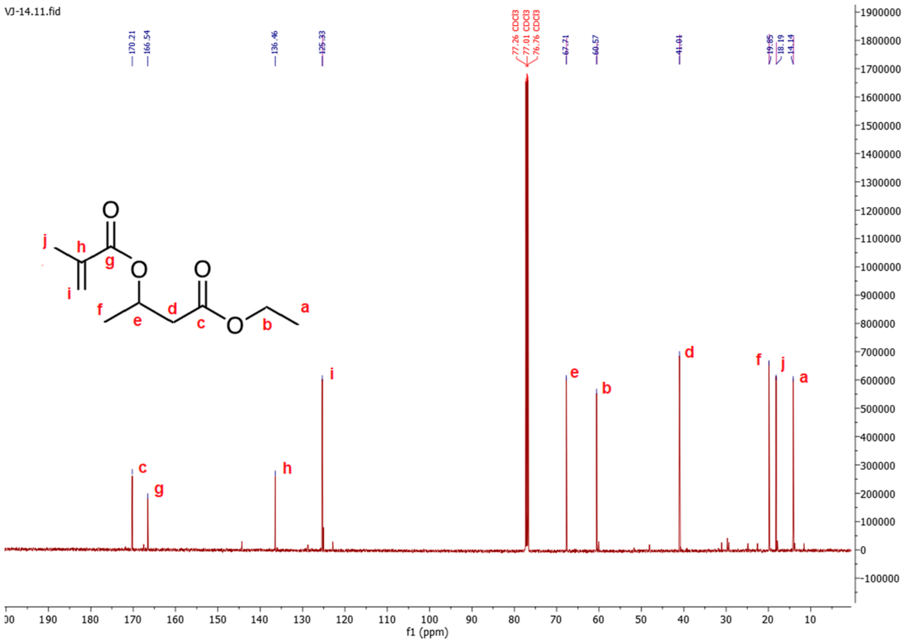

Figure S16. ^{13}C NMR spectrum of methacrylatedethyl 3–hydroxybutyrate (E3HBMMA). ^{13}C NMR ($CDCl_3$, 126 MHz): δ (ppm) 170.21; 166.54; 136.46; 125.33; 67.71; 60.57; 41.01; 19.85; 18.19; 14.14.
